# A Pain in the Buttock

**DOI:** 10.1155/2011/414693

**Published:** 2011-11-29

**Authors:** Zachary C. Landman, Shannon Beres, Michael D. Cabana

**Affiliations:** ^1^Department of Pediatrics, University of California, San Francisco, CA 94117, USA; ^2^Epidemiology and Biostatistics, University of California, San Francisco, CA 94143, USA; ^3^The Institute for Health Policy Studies, University of California, San Francisco, CA 94143, USA

## Abstract

Spondylolysis, a fracture of the pars interarticularis, is a common source back pain in children and adolescents. While the incidence is significantly higher in Asian and Inuit populations, it is never seen in nonambulatory children and is most commonly associated with athletic activities that involve extension or rotational deformity about the spine suggesting a functional component. Given that the associated pain is typically insidious in onset, lacks preceding trauma, and is accompanied by muscular spasm, prompt diagnosis requires a high index of suspicion, familiarity with provocative testing, and knowledge of the appropriate radiographic evaluation. Treatment requires cessation of athletic activity, bracing, and rest for a minimum of four to six weeks, or until symptomatic and radiographic resolution.

## 1. Introduction

Musculoskeletal pain is a very serious concern for young athletes and their parents, coaches, and physicians. Inadequate treatment can lead to discontinuation of athletics entirely [1], increased rates of reinjury [2], and osteoarthritis later in life [3]. It also represents a frequent presenting complaint in primary care practice [4] and carries a significant national financial burden in its treatment [5]. Furthermore, inadequate experience or training in musculoskeletal conditions has been shown to lead to inappropriate referrals, delayed referrals, and suboptimal management [6]. As this case represents, locating the origin of pain in children and adolescents is challenging and must be directed by the history, physical exam, and clinician's index of suspicion.

## 2. Case Presentation

The patient is a 14-year-old Asian-American adolescent female who presented with a chief complaint of left hip pain for several months. The patient described a sharp, nonradiating “7 out of 10” pain in the left buttock and hip, exacerbated with transition from seated to erect, and mildly improved with rest and anti-inflammatory medications. While the pain worsened from an unprovoked fall backwards during an athletic match on the day prior to presentation, it was initially noticed three months prior. The pain was insidious in onset, progressive in nature, and there was no recalled trauma or instigating event. As an active high school soccer athlete, the patient had continued her athletic activities in the interim, using ibuprofen and intermittent rest as needed for pain relief. Further questioning revealed that a similar pain was present between six and three months prior in the right buttock, but has since improved. Past medical history was notable for immune thrombocytopenic purpura. Menarche occurred at age 13 and was regular. The patient was not taking any medications or vitamins. Family history was negative for rheumatologic diseases.

**Figure 1:**
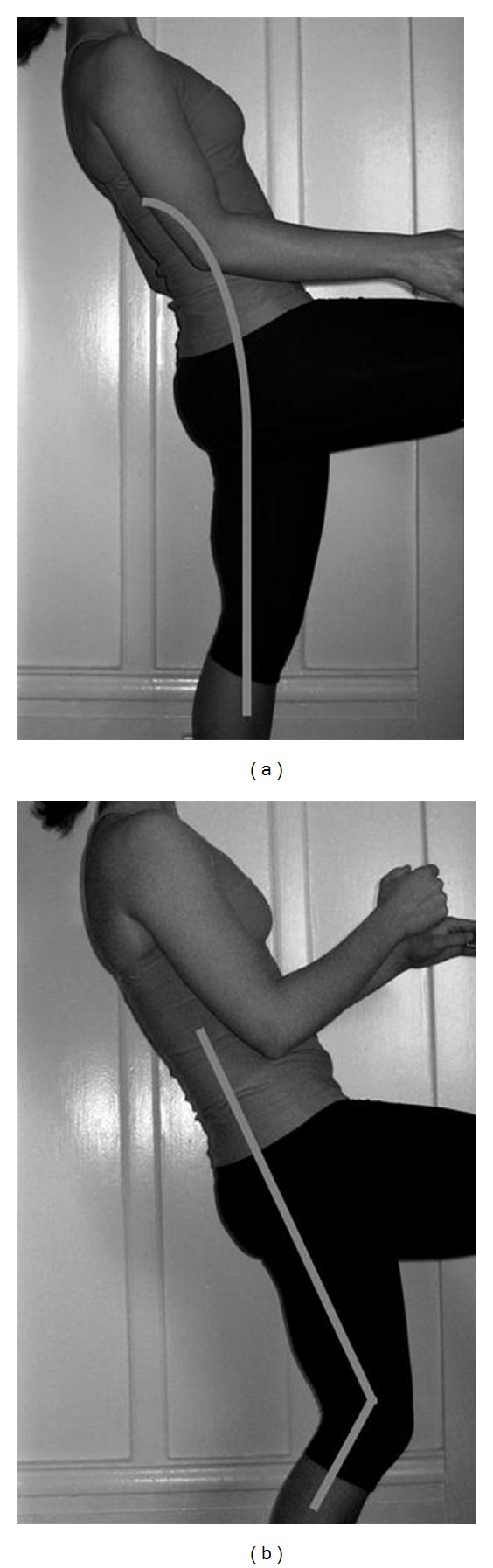
The stork test involves asking the patient to stand on one leg and to extend the low back. Pain indicates possible spondylolysis on the ipsilateral side. When properly done (a), the leg is straight and trunk tilt results from lumbosacral extension. Note that concurrent ipsilateral knee flexion (b) may produce trunk tilt without low back extension and subsequently produce lower test sensitivity.

**Figure 2:**
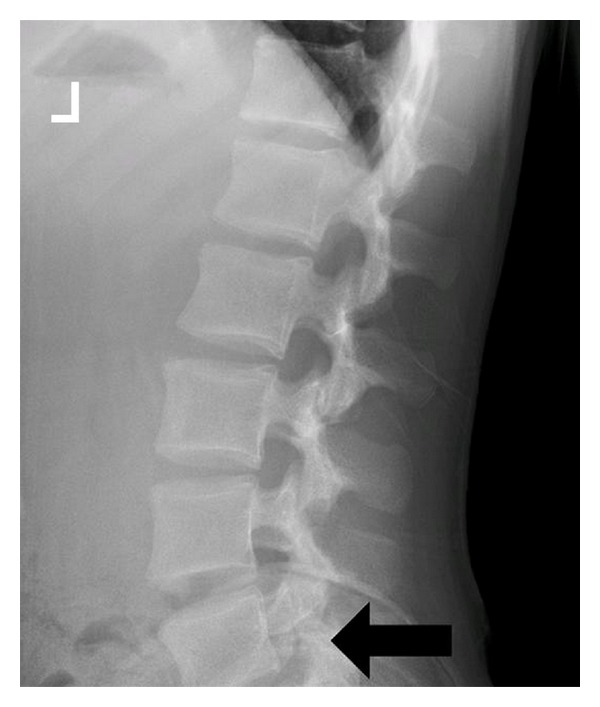
Hyperlucency seen at the L5 pars interarticularis is suggestive of fracture.

On physical exam, her height was 152.5 cm (11th percentile) and weight was 40.6 kg (13th percentile). The vital signs were within normal limits. She was sitting calmly, intermittently grimacing with positional changes. The chest was clear to auscultation bilaterally with no chest wall abnormalities. The abdomen was soft, nontender, and nondistended. Inspection of the spine and lower extremities revealed no erythema, effusions, or signs of trauma. The patient had marked tenderness over the left sacroiliac joint on palpation. Her range of motion was complete and nonpainful at the hips, while active and passive knee extension was limited bilaterally (ROM: 135° to 20°, normal 135°–0°). Provocative tests revealed reproduction of pain with one-leg standing and concordant low back extension (stork test) (Figure 1). Observation of her gait demonstrated an exaggerated hip swing on the left. The neurologic exam was nonfocal and intact with normal strength, reflexes, and tone. Given the positive stork test, coronal and lateral roentgenograms of the spine were obtained, revealing hyperlucency at the L5 pars interarticularis suggestive of bilateral fractures of the pars interarticularis (Figure 2). An MRI was obtained for further evaluation, which demonstrated bilateral L5 pars defects with surrounding bone marrow defects and early sclerosis, confirming the presumed diagnosis. The patient's case was discussed with consultants from the pediatric orthopaedic surgery department who advised outpatient treatment with rest, complete restriction from athletic activities, and a lumbosacral orthotic for a minimum of six weeks. Upon three months followup, the patient's pain had ceased and was advised to slowly discontinue bracing as tolerated.

## 3. Discussion

While this patient initially presented with pain in the hip and buttock, the lack of groin involvement, or limitation of hip motion coupled with tenderness to palpation at the sacroiliac joint on exam suggested a spinal etiology. As such, more provocative testing of the spine revealed pain with lumbar extension and subsequently directed the radiographic examination, ultimately leading to the diagnosis. Spondylolysis, initially characterized by Wiltse and colleagues in 1976, is a defect of the pars interarticularis of the posterior vertebral elements [7]. There is considerable ethnic variation as studies have shown an incidence of 2% in African Americans and 5.9% in Japanese to 54% in Inuits [8]. While there is a strong genetic component, it is never seen in infants or in nonambulatory children, suggesting that upright posture and axial loading are essential. In the pediatric population, spondylolysis is therefore more common in adolescents than younger children (4.4% of six-year olds in contrast to 6.6% of eighteen-year olds) [8]. In contrast to adults, it is a common source of back pain in children, accounting for 47% of all cases by some accounts [9]. Activities that frequently cause hyperextension and/or rotational motions about the spine have much greater incidences of both symptomatic and asymptomatic pediatric spondylolysis. These activities include baseball (23.6%), American football (15.6–30.8%), gymnastics (8.2%–11%), ballet (32%), wrestling (30%), and diving (43%) [8, 10].

Wiltse and colleagues initial postulation that adolescent spondylolysis represented a stress fracture of the pars has been repeatedly supported by epidemiological and biomechanical evidence. The L5 vertebrae is unique in that its forward facing inferior facets articulate with the backward facing sacral facets, resisting sheer forces, and contributing to the stability of the lumbosacral joint. Furthermore, the load on the posterior bony arches during extension is maximally concentrated at the L5 vertebrae. Children and adolescents are at especially high risk when subjected to repeated loads due to their hyperelastic intervertebral disks that are less able to resist sheer stress [8]. In addition, children and adolescents have incompletely ossified pars that are less able to resist repeated loads without eventual failure [10].

As was present in this patient, concurrent or independent muscular spasm or spondylolisthesis (anterior translation of a vertebra upon another) can also cause pain in the buttocks and hips, with or without radicular symptoms. A directed muscular and spine exam with stork test is warranted to help differentiate independent and concurrent sources of pain. The clinician should note the presence of a knee-flexed, hip-flexed gait (Phalen-Dickson sign), hamstring tightness (present in 80% of patients), and tenderness overlying the area. Having the patient stand on one leg and concordantly extend the low back (stork test) will reproduce the pain. Although rare, bowel and bladder function should be reviewed given the potential for cauda equina syndrome in the setting of severe associated spondylolisthesis.

When presented with a case of suspected spondylolysis, classic teaching suggests obtaining AP, lateral, and oblique radiographs of the spine and lumbosacral region. However, since greater than 80% of fractures can be detected with coronal and sagittal films initially and oblique radiographs double the radiation dose to the patient [10], some authors may suggest that the initial evaluation should include only the coronal and sagittal films, and to use focal CT scan, MRI, or oblique views as an adjunct in cases of unclear diagnosis. While serious disability is rare in spondylolysis and follows a generally benign course, it can lead to spondylolisthesis and associated neurologic compromise as well as progressive back pain. Initial treatment includes cessation of athletic activity, rest, use of a lumbosacral orthotic to prevent lumbar extension for a minimum of four to six weeks, and physical therapy directed at improving core strength to help prevent recurrence. Upon resolution of pain, the athlete may gradually return to athletic activity. Spondylolysis is a common cause of low back pain in active children and adolescents and requires a high-degree of suspicion to ensure prompt diagnosis and treatment.
